# General Relationships between Abiotic Soil Properties and Soil Biota across Spatial Scales and Different Land-Use Types

**DOI:** 10.1371/journal.pone.0043292

**Published:** 2012-08-22

**Authors:** Klaus Birkhofer, Ingo Schöning, Fabian Alt, Nadine Herold, Bernhard Klarner, Mark Maraun, Sven Marhan, Yvonne Oelmann, Tesfaye Wubet, Andrey Yurkov, Dominik Begerow, Doreen Berner, François Buscot, Rolf Daniel, Tim Diekötter, Roswitha B. Ehnes, Georgia Erdmann, Christiane Fischer, Bärbel Foesel, Janine Groh, Jessica Gutknecht, Ellen Kandeler, Christa Lang, Gertrud Lohaus, Annabel Meyer, Heiko Nacke, Astrid Näther, Jörg Overmann, Andrea Polle, Melanie M. Pollierer, Stefan Scheu, Michael Schloter, Ernst-Detlef Schulze, Waltraud Schulze, Jan Weinert, Wolfgang W. Weisser, Volkmar Wolters, Marion Schrumpf

**Affiliations:** 1 Department of Biology, Biodiversity and Conservation Science, Lund University, Lund, Sweden; 2 Max Planck Institute for Biogeochemistry, Jena, Germany; 3 Geoecology, University of Tübingen, Tübingen, Germany; 4 Department Animal Ecology, J.F. Blumenbach Institute of Zoology and Anthropology, Georg August University of Göttingen, Göttingen, Germany; 5 Soil Biology Section, Institute of Soil Science and Land Evaluation, University of Hohenheim, Hohenheim, Germany; 6 Department Soil Ecology, UFZ - Helmholtz Centre for Environmental Research, Halle, Germany; 7 Geobotany, Ruhr-University Bochum, Bochum, Germany; 8 Chair of Soil Ecology, Institute of Biology, University of Leipzig, Leipzig, Germany; 9 Institute of Microbiology and Genetic, Georg August University of Göttingen, Göttingen, Germany; 10 Department Animal Ecology, Justus Liebig University Gießen, Gießen, Germany; 11 Department of Biodiversity Research/Systematic Botany, Institute for Biochemistry & Biology, University of Potsdam, Potsdam, Germany; 12 Leibniz-Institute DSMZ - German Collection of Microorganism and Cell Cultures, Braunschweig, Germany; 13 Department Forest Botany and Tree Physiology, Büsgen Institute, Georg August University of Göttingen, Göttingen, Germany; 14 Chair for Soil Ecology, Technische Universität München, München, Germany; 15 Department of Microbial Ecophysiology, University of Bremen, Bremen, Germany; 16 Research Unit for Environmental Genomics, Helmholtz Zentrum München, München, Germany; 17 Max-Planck-Institute for Molecular Plant Physiology, Jena, Germany; 18 Department Terrestrial Ecology, University of Cologne, Zoological Institute, Cologne, Germany; 19 Institute of Ecology, FSU Jena, Jena, Germany; U. S. Salinity Lab, United States of America

## Abstract

Very few principles have been unraveled that explain the relationship between soil properties and soil biota across large spatial scales and different land-use types. Here, we seek these general relationships using data from 52 differently managed grassland and forest soils in three study regions spanning a latitudinal gradient in Germany. We hypothesize that, after extraction of variation that is explained by location and land-use type, soil properties still explain significant proportions of variation in the abundance and diversity of soil biota. If the relationships between predictors and soil organisms were analyzed individually for each predictor group, soil properties explained the highest amount of variation in soil biota abundance and diversity, followed by land-use type and sampling location. After extraction of variation that originated from location or land-use, abiotic soil properties explained significant amounts of variation in fungal, meso- and macrofauna, but not in yeast or bacterial biomass or diversity. Nitrate or nitrogen concentration and fungal biomass were positively related, but nitrate concentration was negatively related to the abundances of Collembola and mites and to the myriapod species richness across a range of forest and grassland soils. The species richness of earthworms was positively correlated with clay content of soils independent of sample location and land-use type. Our study indicates that after accounting for heterogeneity resulting from large scale differences among sampling locations and land-use types, soil properties still explain significant proportions of variation in fungal and soil fauna abundance or diversity. However, soil biota was also related to processes that act at larger spatial scales and bacteria or soil yeasts only showed weak relationships to soil properties. We therefore argue that more general relationships between soil properties and soil biota can only be derived from future studies that consider larger spatial scales and different land-use types.

## Introduction

Very few principles are known that explain the relationship between soil properties and soil biota across large spatial scales and land-use types, as most studies have traditionally focused on small spatial scales [1]. Although these small scale studies provide information about the relationship between soil properties and biota in specific habitats under local conditions, they do not identify the patterns shared among different regions and land-use types. Soil acidity for example influences assemblage turnover in terrestrial snails, but such results are based on a high covariation between geographic position and soil acidity. General relationships between soil properties and biotic responses should therefore be studied after correcting for large-scale effects [2]. Recently, it was emphasized that soil ecologists have identified only few of these unifying principles that can explain patterns in the belowground system over larger spatial scales and across different land-use types [3].

Soil biota play an important role in many ecosystems by ensuring a number of functions such as decomposition and nutrient mineralization [4]. As these ecosystem services are threatened by land-use changes [5], an improved knowledge about the general relationship between soil properties and soil biota over relevant spatial scales and in different land-use types is needed to predict consequences of future changes.

Here, to contribute to such an improved understanding, we performed a comprehensive sampling campaign in differently managed forest and grassland plots in three regions that span a latitudinal gradient of more than 500 km [6]. Using data on abiotic soil properties and soil biota ranging from bacteria to macrofauna we hypothesize that, after extracting the variation that is explained by location and land-use type, soil properties alone will still explain significant proportions of variation in abundance and diversity patterns of soil biota.

**Table 1:** Variables of a) abiotic soil properties, b) soil biota abundance/biomass/concentration and c) soil biota diversity, measurement unit, data range and method.

Variable	Unit	Range	Method
**a) Soil properties**			
Soil pH	NA	3.0–7.4	0.01 M CaCl_2_
Clay content	g/kg	1–670	Pipette method
Total nitrogen	g/kg	1.0–23.9	Elemental analyzer
C/N ratio	NA	8.7–20.5	Elemental analyzer
Nitrate	mg/kg	0.4–235.4	Continuous flow analyser
Ammonium	mg/kg	0.0–8.2	Continuous flow analyser
Plant-available phosphorus	mg/kg	25.9–819.9	Molybdenum blue
**b) Biota abundance**			
Total microbial biomass	nmol/g soil	2.3–139.8	PLFA
Gram-negative bacteria^1^	nmol/g soil	0.1–6.6	PLFA
Gram-positive bacteria^1^	nmol/g soil	0.0–4.5	PLFA
% Acidobact. DNA/tot. bact. DNA^1^	%	0–62	Quantitative PCR
% Acidobact. cDNA/tot. bact. DNA^1^	%	4–16	Quantitative PCR
Bact.cDNA/total DNA ratio^1^	NA	6–44002	Quantitative PCR
Acidobact. cDNA/tot. DNA ratio^1^	NA	5–51257	Quantitative PCR
Saprotrophic fungi 1^2^	nmol/g soil	0.15–12.04	PLFA
Saprotrophic fungi 2^2^	nmol/g soil	0.03–7.00	PLFA
Arbuscular mycorrhiza^3^	nmol/g soil	0.0–7.4	PLFA
Yeasts, colony forming units^4^	CFU/g soil	60.0–115500.0	Cultivation experiments
Yeasts, biomass^4^	mgC/g soil	0.001–1.18	Cultivation experiments
Fungal/bacterial ratio	NA	1.0–4.7	PLFA
Acari^5^	ind/m^2^	1273–283769	Kempson extraction
Collembola^5^	ind/m^2^	891–153718	Kempson extraction
Lumbricidae^5^	ind/m^2^	0–716	mustard sol./hand sorting
Myriapoda^5^	ind/m^2^	0–3220	Kempson extraction
Free amino acids	nmol/kg	90.2–1524.6	HPLC
**c) Biota diversity**			
Yeasts^6^	Shannon index	0.0–1.9	Incubation
Extracellular proteins^7^	Shannon index	0.5–1.6	Chromatography
Lumbricidae^8^	species/plot	0–6	Kempson extraction
Myriapoda^8^	species/plot	0–11	Kempson extraction

Soils are classified according to [44], for further details see material and methods or supporting information S1.

Classification of groups for follow-up models after obtaining a significant overall model for the relationship between abiotic soil properties and soil biota abundance:

1 bacteri.

2 total biomass of saprotrophic fungi.

3 arbuscular mycorrhizal fungi.

4 yeasts.

5 soil fauna or diversity.

6 yeasts.

7 extracellular proteins.

8 soil fauna.

Abbreviations: Acidobact. = Acidobacteria, bact. = bacteria, tot. = total.

## Materials and Methods

### Study Regions

The sampling campaign took place in the framework of the “biodiversity exploratories project” and full details of the design are given in [6]. Briefly, the three regions are the ‘Schwäbische Alb’ in the low mountain ranges of south-western Germany, the ‘Hainich-Dün’ in central Germany, and the ‘Schorfheide-Chorin’ in the lowlands of north-eastern Germany. Soils in the Schwäbische Alb Exploratory are dominated by Cambisols for forest and Leptosols for grassland sites. Soils in the Hainich-Dün Exploratory are dominated by Luvisols for forest and Stagnosols for grassland systems. Soils in the Schorfheide-Chorin Exploratory are dominated by Arenosols for forest and Histosols and Gleysols for grassland systems. Annual average precipitation and temperature are: Schwäbische Alb 938–963 mm & 6.5–8.0°C; Hainich-Dün 750–800 mm & 6.5–7.5°C and Schorfheide-Chorin 520–600 mm & 8.0–8.4°C [6]. In each exploratory three land-use types were studied in forests (unmanaged beech forests, and managed forests of beech and conifers) and grasslands (meadows, pastures and mown pastures) with three replicates per type. Due to the incomplete dataset of one forest and one grassland plot from Hainich-Dün, we excluded these sites from the statistical analyses. Field work permits were issued by the following state environmental offices: Regierungspräsidium Tübingen (Schwäbische-Alb), Thüringer Landesverwaltungsamt (Hainich-Dün) and Landesumweltamt Brandenburg (Schorfheide-Chorin). Further details on the regions, their properties and the field permits are provided in Fischer et al. [6].

**Table 2:** Results of distance-based linear models testing for relationships between a) sampling location, land-use type or abiotic soil properties and soil biota abundance or diversity patterns in marginal tests that relate each predictor group individually and in sequential tests that first extracted variation from location and land-use type (R^2^ = 0.58) and b) abiotic soil properties and abundance or diversity patterns of individual soil biota groups in sequential tests that were first fitted for location and land-use type.

	Abundance		Diversity	
a)	R^2^	P	R^2^	P
*Marginal tests*				
Location	0.16	0.002	0.14	0.001
Land-use	0.42	<0.001	0.18	0.024
Soil properties	0.46	<0.001	0.28	0.003
*Sequential tests*				
Soil properties	0.10	0.036	0.20	0.009
**b)**	**R^2^**	**P**	**R^2^**	**P**
Bacteria	0.10	0.180	NA	
Yeast	0.12	0.363	0.15	0.305
AM fungi	0.23	<0.001	NA	
Saprotrophic fungi	0.22	<0.001	NA	
Soil fauna	0.09	0.018	0.24	0.013
Extracellular proteins	NA		0.21	0.076

### Sampling

At each sample location, soil was sampled from five points in a 20×20 m area to obtain a composite sample for the analysis of abiotic soil properties (Table 1a). After removal of aeromorphic organic layers, mineral soils were sampled horizon-wise down to the parent material using a motor driven auger with a diameter of 8.3 cm (Eijkelkamp, Giesbeek, The Netherlands). The organic soils of the Schorfheide-Chorin grassland were sampled using a split-tube sampler with a diameter of 5.6 cm. In this study, only results from the upper mineral soil horizon (A, E or H horizon) were considered. The following abiotic soil properties that affect soil biota at small spatial scales [9] were used in our analysis: soil pH, clay content, total nitrogen, C/N ratio, nitrate, ammonium and plant-available phosphorus concentrations (Table 1a).

**Figure 1:**
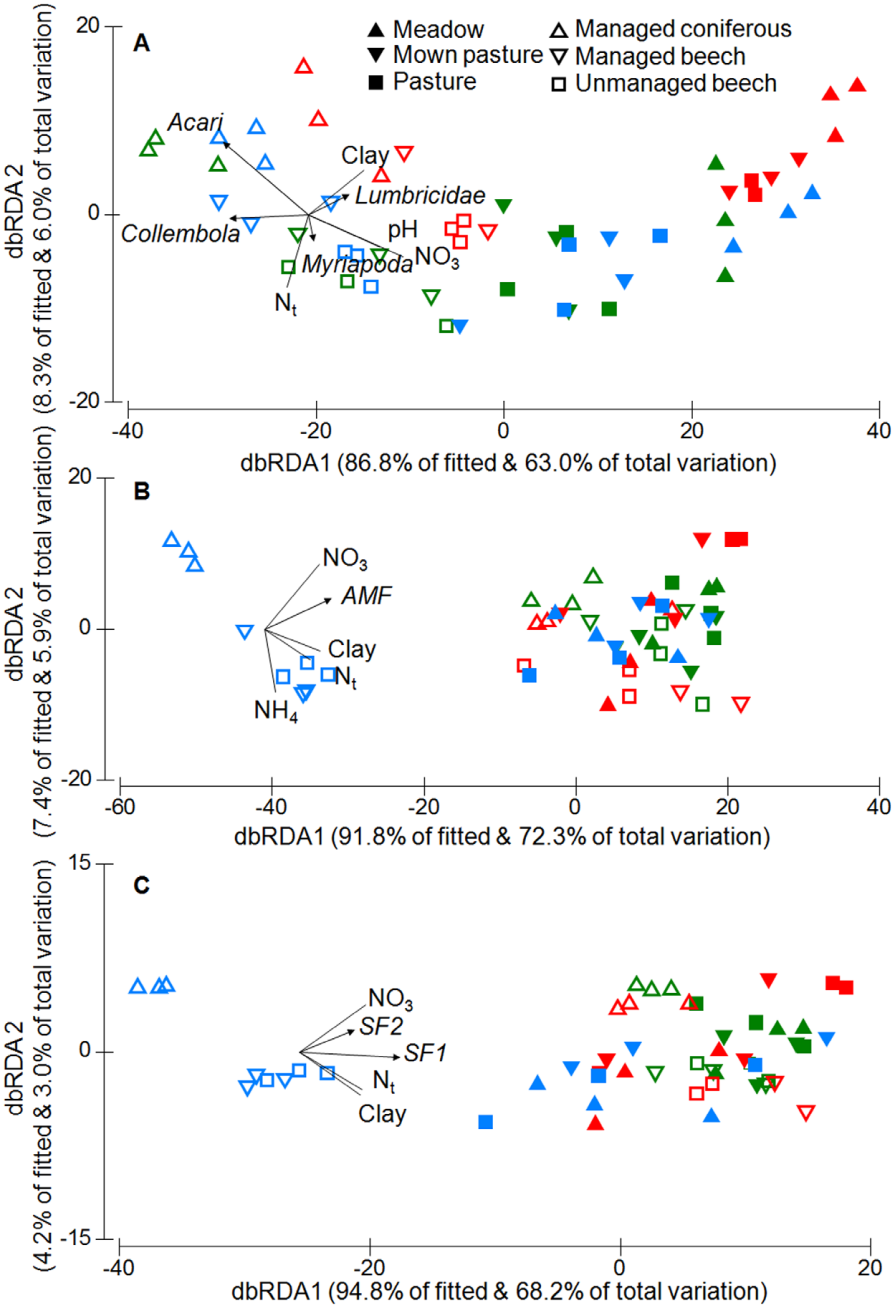
The relationship between soil properties and abundance/biomass of soil biota. Distance-based RDA triplot showing the relationship between soil properties and A) the abundance of soil fauna, B) the biomass of arbuscular mycorrhiza (AMF) and C) the biomass of saprotrophic fungi (SF1 & 2) in three study regions (colours: blue, Schorfheide-Chorin; red, Hainich-Dün; green, Schwäbische-Alb) and six land-use types (symbols). Vectors for soil properties are only shown if multiple correlation coefficients >0.4 (clay, clay content; NH_4_, ammonium content; NO_3_, nitrate content; N_t_, total soil nitrogen & pH, soil pH).

Soil arthropods (Acari, Collembola and Myriapoda) were sampled by collecting one soil core (diameter 20 cm, depth 5 cm) in grasslands and two soil cores (diameter 5 cm for Acari and Collembola, diameter 20 cm for Myriapoda, depth 5 cm) in forests at each sampling location. Soil fauna in forest plots was sampled before the organic layer was removed and all soil cores were extracted using a modified heat extraction system [7]. Earthworms were hand sorted from two large soil cores in grassland plots (diameter 20 cm; depth 10 cm) or extracted from a 50 cm^2^ area using mustard solution as expellant [8]. All soil fauna abundances are expressed as individuals per m^2^ (for further details see supporting information S1).

**Figure 2:**
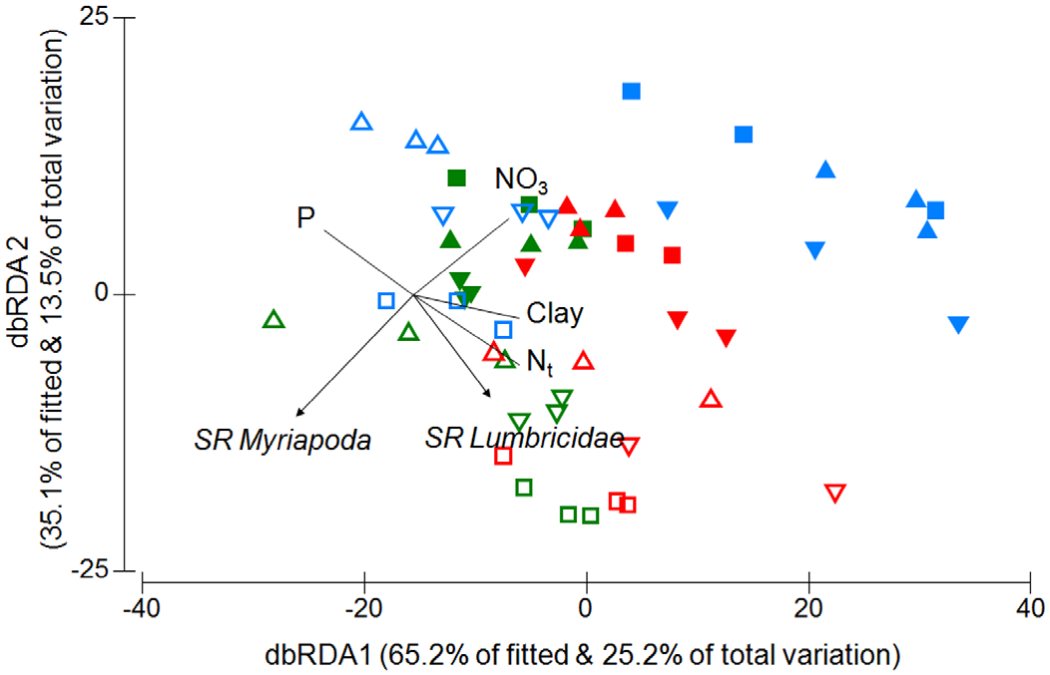
The relationship between soil properties and diversity of soil biota. Distance-based RDA triplot showing the relationship between the diversity of soil fauna (arrows) and soil properties (lines) in three study regions (colours: blue, Schorfheide-Chorin; red, Hainich-Dün; green, Schwäbische-Alb) and six land-use types (symbols). Vectors for soil properties are only shown if multiple correlation coefficients >0.4 (clay, clay content; NO_3_, nitrate content; N_t_, total soil nitrogen & P, plant-available phosphorous). For a legend to the symbols please refer to Fig. 1.

The data on soil biota abundances, concentrations and biomasses include microorganisms (gram-negative and gram-positive bacteria, Acidobacteria, saprotrophic fungi (SF), arbuscular mycorrhizal fungi (AMF), cultivable yeast fungi and free amino acids) and soil meso- and macrofauna (Acari, Collembola, Lumbricidae and Myriapoda; Table 1b). The data on diversity of soil biota include the Shannon index of yeasts, and of extracellular proteins in soil originating from viruses, archaea, bacteria, fungi, other unicellular eukaryotes, plants or animals and the species richness of earthworms and myriapods that were identified to species level (Table 1c; for further details see supporting information S1).

### Statistical Analyses

We analysed the relationship between abiotic soil properties (Table 1a) and patterns in soil biota abundances (Table 1b) or diversity (Table 1c) using distance based linear models [10]. Prior to analyses, we defined three indicator groups that included different subsets of individual predictor variables: (A) location, as continuous variable (X and Y geographic coordinates) assuming a linear gradient in large scale differences between regions (as for example average annual precipitation declines from south to north) [11], (B) land-use type, as binary coded variables reflecting one of six land-uses (unmanaged beech forests, managed forests of beech and conifers, meadows, pastures and mown pastures), and (C) soil properties, as the seven continuous variables in Table 1a. Variables that were measured at different scales were normalized as part of a standard procedure in distance-based linear models [12]. In a first set of analyses separate models were used to test for the individual relationship between each indicator group (A)–(C) and either multivariate soil biota abundance (Table 1b) or diversity (Table 1c) patterns. This approach provides the proportion of explained variation in similarities between sites based on soil biota abundance or diversity by each indicator group. Second, additional distance based linear models were used to first fit location and land-use type to soil biota abundance or diversity, thereby extracting variation that originates from large scale differences among sampling locations and land-use type. The remaining variation in soil biota abundance or diversity was then tested in the same models for relationships with abiotic soil properties. This approach indicates if abiotic soil properties were related to soil biota after accounting for sampling location and land-use type. This approach is conservative and rather underestimates the amount of explained variation. After obtaining a significant result in these multivariate models that account for the non-independence between soil organism groups, sub-groups of abundance or diversity variables (Table 1, groups for follow-up models) were tested with identical models to identify the importance of individual soil properties for explaining variation of groups of soil biota. While for soil properties Euclidean distances were used, as joint absences were meaningful, Bray-Curtis distances were used for soil biota to avoid that joint absences contribute to similarities between sites [13]. All distances were calculated based on square-root transformed data and P-values were obtained from 9999 permutations. Distance-based redundancy analysis was used to perform ordinations of the fitted values from distance-based linear models [12] and to show the relationship between important soil properties and individual soil organism variables. All models were calculated in Primer-E [14].

## Results

### Abundances

Location, land-use type and abiotic soil properties explained significant proportions of variation in the abundance of soil biota if each predictor group was analyzed separately (Table 2a). Location and land-use type together explained more than 58% of the variation in overall soil biota abundance. After accounting for this variation, abiotic soil properties still explained a significant proportion of variation in abundance data for soil biota (Table 2a). Together, land-use type, location and soil properties explained more than 68% of the variation in soil biota abundances. Testing individual taxonomic groups indicated that abundance patterns in arbuscular mycorrhizal fungi (AMF), saprotrophic fungi as a whole (SF) and soil fauna were significantly related to soil properties independent of location or land-use type (Table 2b). In contrast, biomass variables for bacteria and yeasts were not significantly related to soil properties. Mites and Collembola were generally more abundant in forest compared to grassland soils, with the highest mite abundances in managed conifer and beech forests (Fig. 1a). Mites and to a lesser extent Collembola were most abundant in soils with low pH values and nitrate concentration after accounting for location and land-use type. The biomass of AMF and SF was lowest in forest habitats in the Schorfheide-Chorin region (Fig. 1b), but no other differences in fungal biomass were observed between the analysed regions or land-use types. Both groups of fungi (AMF and SF) were positively related to nitrate and nitrogen concentration in soils (Figs. 1b, c).

### Diversity

Location, land-use type and abiotic soil properties explained significant proportions of variation in the diversity of soil biota if analyzed separately (Table 2a). Location and land-use type together explained more than 33% of the variation in soil biota diversity. After accounting for the relationship to sampling location and land-use type, abiotic soil properties still explained a significant proportion of variation in soil biota diversity (Table 2a). Taken together, the three indicator groups explained more than 53% of the variation in soil biota diversity. Testing diversity values suggests that macrofauna diversity was significantly related to abiotic soil properties independent of location or land-use type (Table 2b). In contrast, diversity of extracellular proteins and soil yeasts were not significantly related to soil properties. Myriapod assemblages had higher species richness in most forest soils, with particularly low richness in grasslands in the Schorfheide-Chorin region (Fig. 2). Earthworms were most diverse in beech forest soils of the Hainich-Dün and Schwäbische-Alb region. After accounting for the observed differences between locations and land-use types, myriapod species richness was negatively related to soil nitrate concentration. Earthworms in contrast had higher species richness in soils with high total nitrogen concentration and clay content, but low concentrations of plant-available phosphorous.

## Discussion

It has been recognised that the role of environmental variability as predictor of organism diversity and abundance varies with the scale of ecological studies [1]. However, in soil biology we are only recently beginning to understand the relationship between abiotic and biotic soil characteristics from small to larger spatial scales and across different land-use types [15]–[19]. Here we show that after accounting for heterogeneity resulting from large scale differences among sampling locations and land-use types, soil properties still explain significant proportions of variation in soil biota abundances and diversity.

Land-use is known to affect belowground communities, and more intensively managed soils often contain lower fungal biomass [20]. The lowest fungal biomass in our study was observed in forest plots of the Schorfheide region, which were the sampling locations with the most acidic soils (mean pH Schorfheide = 3.2 vs. Alb = 5.1 & Hainich = 4.9) and the lowest clay content (mean Schorfheide = 16 g/kg vs. Alb = 448 g/kg & Hainich = 343 g/kg). The acidity and dominance of sandy soils is known to contribute to low actinomycete biomass [21]. Although low soil pH is considered to be favourable for development of fungi [22], forest plots of the Schorfheide region showed decline in SF and AMF biomass. Interestingly, this sampling location has the lowest average annual precipitation (520–600 mm) and highest average temperature (8.0–8.4°C). This contrasts the opinion that Fungi are generally more abundant during the drought stress than soil prokaryotes, except for actinomycetes, as they can sustain ultra-low (≤0.8) water activity [23]–[24]. However, responses of particular groups of fungi to low precipitation and soil acidity differed from the response of the fungal community as a whole. Specifically, soil yeasts were more abundant in forest plots in the Schorfheide (see also [25]). The ability of soil yeasts to survive in sandy soils due to production of exogenic polysaccharide capsules has been demonstrated previously [26]. In contrast to forest sites, grasslands in the Schorfheide were not characterized by particularly low fungal biomasses. This study provides evidence that effects of soil acidity and texture on microbial communities might also depend on the type of land use (grassland versus forest). A comparison of abiotic soil properties between grasslands in the different regions supports the assumption that sandy, acidic soils contributed to the low fungal biomasses in forests. Grassland sites in the Schorfheide were not nearly as acidic or low in clay content compared to forest habitats in the region (Schorfheide grassland pH = 7.0 vs. Alb = 6.4 & Hainich = 6.8 and Schorfheide grassland clay content = 255 g/kg vs. Alb = 377 g/kg & Hainich = 468 g/kg).

The abundances of different soil fauna groups showed contrasting relationships with location and land-use type. The highest densities of mites, Collembola and Myriapoda were observed in forest ecosystems, but there were more earthworms in grassland plots. Earthworms were generally abundant in temperate grasslands and may have benefited from resource additions (fertilization) in grasslands [27] or from the generally higher pH values in grassland soils (pH grassland = 6.7 vs. pH forest = 4.4). The high abundance of Collembola and mites confirms their preferences for leaf litter layers of temperate forest ecosystems [27]–[28].

The observed relationships between abundances and diversities of soil biota and sampling locations or land-use types support previous studies. However, at least three patterns could be established that describe more general relationships between soil properties and abundance or diversity of soil biota across sampling locations and land-use types:

1. The biomass of AMF was positively related to nitrate and nitrogen content in soils. Mosse and Phillips [29] hypothesized that plants allocate more carbon to mycorrhizal fungi at locations where nitrogen is limiting and therefore predicted a decline in mycorrhizal biomass if nitrogen availability increases [30]. Indeed, a meta-analysis synthesising results from fertilization experiments showed a negative impact of nitrogen fertilizers (including ammonium nitrate) on mycorrhizal abundance [31] and fungal biomass generally declines with management intensity in grasslands [32]. In our study we found a positive relationship between nitrate concentration and AMF biomass across a range of forest and grassland soils. Kooijman et al. [21] suggested that N mineralization is positively related to fungal biomass and higher fungal biomass in our soils may have contributed to the high nitrate content in our spring sampling, as plants may not have taken up most of the available nitrogen at this date. However, since we measured nitrate concentrations only in spring we cannot conclude on effects of temporal variation in N availability on soil biota during the whole year [33].
2. The abundance of mesofauna and myriapod species richness were negatively related to nitrate content in forest and grassland soils. Nitrogen deposition leads to higher nitrate and ammonium content in forest [34] and grassland [35] soils and the application of synthetic fertilizers is known to affect the abundance and diversity of soil mesofauna negatively in agroecosystems [20], [36]. However, in forests and grasslands the relationship between soil mesofauna and nitrate addition differed among studies, showing either no [37]–[38], weak negative [39], strong negative [40] or even positive [41] relationships.
3. The species richness of earthworms was positively correlated with clay content of soils across sampling locations and land-use types. Many earthworm species benefit from high clay contents since they can digest the carbon and nitrogen resources stored in clay-rich aggregates [42]. The higher earthworm diversity can therefore be explained by the preferences of many earthworm species for soils with high clay contents.

Our study demonstrates that abundance and diversity patterns of fungi and soil fauna relate to soil properties in a general way. However, the weak relationship between soil properties and abundance patterns in bacteria or soil yeasts and diversity of yeasts and extracellular proteins over larger spatial scales should further caution extrapolation of results from small scale studies to larger spatial scales or different land-use types. We therefore emphasise that comprehensive field studies, in which soil biota and additional important soil properties (e.g. salinity [43]) are analysed with standardized methods and over larger spatial scales are essential for a better understanding of unifying principles in soil biology and further emphasize the need for manipulative studies that focus on explaining the different response patterns by fungi and soil fauna versus bacteria.

## Supporting Information

Supporting Information S1 Detailed description of methods to measure abiotic soil properties and soil biota.
(DOC)
