# Supplementary material for: General Relationships between Abiotic Soil Properties and Soil Biota across Spatial Scales and Different Land-Use Types
Source: PLoS One. 2012 Aug 22;7(8):e43292. doi: 10.1371/journal.pone.0043292 (PMC3425568; doi:10.1371/journal.pone.0043292)
Supplement: Supporting Information S1 — Detailed description of methods to measure abiotic soil properties and soil biota. (DOC) [file pone.0043292.s001.doc]

**Supporting information S1**

**Detailed Methods**

*Abiotic soil properties (ammonium, nitrate, plant available phosphorus, total nitrogen, C/N ratio, soil pH, clay content)*

Fresh soil (<2 mm) and 0.01 M CaCl_2_ were used to extract N-NH_4_^+^ and N-NO_3_^-^ (1:10, w/v) (Houba et al. 1986). Concentrations of extractable nitrogen were determined using a San^++^ continuous flow analyser (Skalar, Breda, The Netherlands). All other abiotic soil analyses were done with air dried soil (<2 mm). Plant-available inorganic P (P_i_) was extracted using air dry soil (<2 mm) and 0.5 M NaHCO3 which was adjusted to a pH of 8.5 with 1 M NaOH (Hedley et al. 1982; modified after Kuo 1996, Olsen et al. 1954). P_i_ concentrations in the NaHCO_3_ extracts were determined with a continuous flow analyzer (Bran+Luebbe, Norderstedt, Germany) using the molybdenum blue method (Murphy & Riley 1962). Ground soil samples were taken for total nitrogen analysis by dry combustion (Vario Max, Elementar Analysensysteme GmbH, Hanau, Germany). The C/N ratio was calculated by dividing organic carbon by total nitrogen concentrations. Soil pH was measured with a glass electrode in a suspension of soil and 0.01 M CaCl_2_ (1:2.5 ratio). Determination of clay contents was performed according to Schlichting and Blume (1966).

*Soil biota, microbes (total microbial biomass, gram-negative and gram-positive bacteria, arbuscular Mycorrhiza, saprotrophic fungi, fungal/bacterial ratio, Acidobacteria, yeasts, total and free amino acids)*

To determine microbial biomass, phospholipid fatty acid analysis (PLFA) was performed on frozen (-80°C) and subsequently freeze-dried soil samples. PLFA extractions were done using a modified Bligh and Dyer (1959) method. Briefly, 2 g freeze-dried sample were extracted twice in a chloroform-methanol-citrate buffer (1:2:0.8), followed by overnight phase separation. Fatty acids in the organic phase were then separated using a silica-bonded phase column (SPE-SI; Bond Elut 3CC, 500 mg, Varian Inc.) to remove glyco lipids and neutral lipids. The polar lipids were converted to fatty acid methyl esters by mild alkaline methanolysis. Methyl-esterfied fatty acids were then analyzed using a Hewlett-Packard 6890 Gas Chromatograph equipped with a DB-5ms arylene phase column (0.25 μm internal diameter by 0.25 μm film thickness by 60 m length, Agilent Technologies), and interfaced to an Agilent 5973 mass selective detector. Peak areas of each lipid were converted to nmol g soil^-1^ using an internal standard (19:0 nonadecanoic methyl ester). The total nmol lipid g dry soil^-1^ (sum of all lipids present, 20 carbons or less in length) was used as an index of microbial biomass (Vestal and White 1989; Hill et al. 1993; Zelles et al. 1992; Frostegård and Bååth 1996). Individual lipids were used to indicate broad groups of the microbial community: an average of monounsaturated lipids for Gram negative bacteria (Wilkinson et al. 2002); an average of branched lipids for Gram positive bacteria (Wilkinson et al. 2002); 16:1ω5c for arbuscular mycorrhizal fungi (AMF; Balser et al. 2005) and 18:2ω6,9c for saprotrophic fungi (SF; Balser et al. 2005). The ratio of fungal lipids to bacterial lipids was used to indicate the fungal to bacterial ratio (Frostegård and Bååth 1996).

To determine the percentage of acidobacterial DNA per total bacterial DNA, the percentage of acidobacterial cDNA per total bacterial cDNA, and the ratio of bacterial as well as acidobacterial cDNA to DNA, in brief, genomic DNA and RNA were extracted from soil samples, cDNA was synthesised from RNA, and 16S rRNA gene copy numbers of Bacteria and Acidobacteria in all samples were measured using quantitative (q) PCR. Percentages and ratios were then calculated from qPCR output.

Genomic DNA was extracted using the PowerSoilTM DNA Isolation Kit (MoBio Laboratories, Solana Beach, CA) according to the protocol provided by the manufacturer. Yield and quality of the extracts were verified by standard agarose gel electrophoresis and UV/Vis spectroscopy (NanoDropTM ND-1000, Peqlab, Erlangen, Germany).

RNA was extracted with a protocol for simultaneous extraction of DNA and RNA from 2 x 0.6 g of soil using bead beating in the presence of sodium phosphate and sodium dodecyl sulphate (Henckel et al. 1999). After centrifugation, the aqueous supernatant containing the nucleic acids was extracted with equal volumes of phenol-chloroform-isoamyl alcohol [PCI, 25:24:1 (vol/vol/vol), Sigma-Aldrich, Steinheim, Germany] and chloroform-isoamyl alcohol [CI, 24:1 (vol/vol), Sigma-Aldrich]. After precipitation of nucleic acids with two volumes of polyethylene glycol solution (Griffiths et al., 2000) and centrifugation at 20 000 x g and 4°C for 90 min, nucleic acid pellets were washed once with 70% ethanol and resuspended in 100 ml Elution Buffer (Qiagen, Hilden, Germany), pH 8.5. RNA was prepared from the primary extracts by digestion of co-extracted DNA with RQ1 RNase free DNase I (Promega, Mannheim, Germany) and subsequent re-extraction with PCI and CI as described above. Standard agarose gel electrophoresis served to verify the quality of extracted total nucleic acids and RNA preparations. RNA yields were determined by UV/Vis spectroscopy (NanoDropTM ND-1000, Peqlab, Erlangen, Germany). Complete removal of DNA from the RNA extracts was verified by PCR targeting 16S rRNA genes, using primers 27f (5’-AGAGTTTGATCCTGGCTC AG-3’; Edwards, 1989) and 907r (5'- CCG TCA ATT CCT TTR AGT TT -3'; Muyzer, 1995). The 50 µl reaction mixture contained 1 x PCR Buffer (Applied Biosystems, Carlsbad, CA), 1.5 mM MgCl2 (Applied Biosystems), 50 μM of each dNTP (GE Healthcare, [Little Chalfont](http://en.wikipedia.org/wiki/Little_Chalfont), [UK](http://en.wikipedia.org/wiki/United_Kingdom)), 0.5 μM of each primer, 1 U AmpliTaq DNA-Polymerase (Applied Biosystems), 0.2 mg ml-1 Bovine Serum Albumin (BSA, Roche, Risch, Switzerland), and 20-100 ng of DNA template. The PCR thermal profile included an initial denaturation step at 94°C for 3 min, 25 cycles of 30 s deanturation at 94°C, 45 s primer annealing at 52°C and 60 s extension at 72°C. The final extension step at 72°C was carried out for 7 min. Synthesis of cDNA from RNA extracts was conducted using the ImProm-IITM reverse transcription system (Promega, Madison, WI, USA).

The abundance of Acidobacteria 16S rRNA genes and transcripts was determined by quantitative PCR with group-specific primer 31f (5´-GATCCTGGCTCAGAATC-3´; Barns et al. 1999) and universal primer 341r (5´-CTGCTGCCTCCCGTAGG-3´; Muyzer et al. 1993). For comparison, the total fraction of eubacterial DNA was quantified with the universal primers 341f (5´-CCTACGGGAGGCAGCAG-3´; Muyzer et al. 1993) and 518r (5´-CCGCGGCTGCTGGCAC-3´; Lane 1991). Real-time PCR reactions were performed in an iCyclerQTM Multi-Color Real Time Detection System (Bio-Rad, Hercules, CA) using the iQ SYBR Green Supermix (Bio-Rad). 10 ng of DNA were used in a reaction volume of 25 µl and each determination was run in triplicate. Thermal cycling included an initial denaturation step at 94°C for 3 min, 35 cycles of 30 s denaturation at 94°C, 30 s primer annealing at 59°C for Acidobacteria and 60°C and for Bacteria, respectively, and 30 s extension at 72°C. Following each assay melt curve analysis was conducted to verify product specificity. For calibration of the real-time PCR measurement, almost full length 16S rRNA gene fragments of *Edaphobacter modestus* DSM 18101T were employed. Standard concentrations ranged from 109 to 102 copies per reaction. Copy numbers were calculated according to Ritalahti et al. (2006).

To analyse yeasts, soil samples were placed in 50 ml plastic tubes, suspended (w/v) 1:5, 1:10, and 1:20 in sterile water and shaken on an orbital shaker at 200 rpm for 1 hour. Soil from one plot was analysed in five replicates (sub-samples) and each of the replicates was plated in triplicates. An aliquot of 0.15 ml was plated on the surface of solid media. Acidified glucose-yeast extract-peptone agar (GPYA) was used for cultivation experiments (Yurkov et al. 2011). Plates were incubated at room temperature for 2-3 days and kept at lower temperatures (6-10°C) to prevent fast development of moulds. Plates were checked after 7, 14 and 21 days of incubation. For each sub-sample, yeast quantity was calculated as CFU (colony forming units) per gram of soil at natural humidity. Yeast biomass (g of Carbon / g of soil) was calculated as a mean C content per cell from the yeast quantities (CFU/g) using the average cell volume determined from the range of 33–100 µm^3^ (Bryan et al. 2010) and the approximate cell density 1 g/mL (van Veen & Paul 1979; Bakken & Olsen 1983; Bryan et al., 2010).

To analyse free amino acids, soil samples were sieved (mesh width 5 mm) to remove stones and roots and used for analyses of free amino acids. 40 g of fresh soil were weighed and mixed for 10 min with 40 ml of 1 mM CaCl_2_. The mixture was filtered through a fluted filter (185 mm, Whatmann Schleicher Schuell, Dassel, Germany). After 1 h the collected filtrate was filtered again through a glass fibre filter (pore size 1 µm, Pall Life Science, Port Washingtion, NY, USA) and subsequently through a filter for sterilization (Sarstedt Filtropur S 0.2 µm, Nümbrecht, Germany). The volume of the filtrate was determined. The filtrate was freeze-dried and the pellet was dissolved in 0.5 ml double deionized H_2_O yielding a concentrated soil extract. Amino acids were analysed by HPLC (Pharmacia/LKB, Freiburg, Germany) using fluorescent o-phthaldialdehyde (OPA) pre-column derivatization according to Riens et al. (1991). o-Phthalaldehyde in conjunction with a thiol reagent reacts with primary amine groups to form highly fluorescent isoindole products. 20 µl of the concentrated soil solution were derivatised for 1 min at 15°C with 20 µl of 10 mM o-phthaldialdehyde solution (60% methanol; 0.7 M borate buffer, pH 10.5; 0.8% mercaptoethanol). Subsequently, 20 µl of the derivatized solution were separated on a column (RP 18 endcapped column, Merck, Darmstadt, Germany) with gradient of 18 mM phosphate buffer, pH 7.1 and acetonitril. Peaks were detected by fluorescence (fluorescence detector, LKB/Pharmacia, Freiburg, Germany) at the excitation wavelength of 330 nm and an emission wavelength of 450 nm. Blanks were run treating 1 mM CaCl_2_ solution in same way as the samples. Amino acid standards (Sigma-Aldrich, München, Germany) were measured in the same way and linear calibration curves (0.1-20 µM) were produced for each amino acid. The primary amino acids aspartate, glutamate, asparagine, serine, histidine, glutamine, glycine, threonine, arginine, alanine, gaba, tyrosine, valine, methionine, isoleucine, phenylalanine, leucine, and lysine were detected. Detection of the secondary amino acid proline was not possible in this assay, and the concentration of cysteine was too low for detection. The concentrations of amino acids in the soil solutions (µM) were calculated from peaks using the integration and calculation program PeakNet 5.1 (Dionex, Idstein, Germany).

*Soil biota, extracellular proteins (viruses, archaea, bacteria, fungi, unicellular eukaryotes, plants and animals)*

Proteins were extracted from frozen (-20°C) soil using an extraction buffer of 50 mM TrisHCl pH 8, 150 mM CaCl_2_, 1% insoluble PVPP and protease inhibitor cocktail (Complete Tabs, Roche). Protein was subsequently precipitated using five volumes of ice cold acetone. Protein pellets were resuspended in 6 M urea, 2 M thiourea, pH 8, and digested to peptides using trypsin as described previously for organic material extracted from soil particles (Schulze, 2005a). Digested protein was subsequently analyzed by liquid-chromatrography-coupled tandem mass spectrometry on an LTQ-Orbitrap mass spectrometer (Schulze 2005b). Collected fragment spectra were matched against the non-redundant NCBI database of protein sequences using Mascot (Matrix Sciences, UK). Peptide sequences were assigned to proteins based on the Mascot algorithm. For each identified protein, the taxonomic group of the assigned organism was retrieved using the NCBI Taxonomy Browser. Due to sequence conservation in related species, taxa were differentiated only on a coarse hierarchical level. Thus, we distinguished the organisms of protein origin as viruses, archaea, bacteria, fungi, unicellular eukaryotes, plants, and animals.

*Soil fauna (Acari, Collembola, Lumbricidae and Myriapoda)*

Soil arthropods (Acari, Collembola and Myriapoda) in grasslands were sampled by collecting one soil core (diameter 20 cm, depth 10 cm) from each plot and fauna was extracted using a modified heat extraction system (Kempson et al. 1963). Earthworms were hand sorted from two large soil cores (diameter 20 cm; depth 10 cm) per plot. Soil fauna in forests was sampled from the litter layer and upper 5cm of soil by taking two soil cores (diameter 5 cm for Acari and Collembola, diameter 20 cm for Myriapoda) from each plot, fauna was extracted by heat (Kempson et al. 1963), animal counts of both layers and subsamples were pooled. Earthworms were extracted on each forest plot using mustard solution as expellant (Eisenhauer et al. 2008). The solution was prepared by soaking 100 g of mustard flour (Semen Sinapis plv., Caesar & Loretz GmbH, Hilden, Germany) in 10 l of water overnight. For the extraction, an area of 50 cm^2^ was confined using a metal frame, the litter material was removed and sorted for earthworms by hand, 5 l of mustard solution was applied on the soil in the beginning of the extraction and additional 5 l after 15 minutes. For 30 minutes in total all surfacing earthworms in the extraction area were collected.

**References**

Bakken, L. R. and Olsen, R. A. 1983. Buoyant densities and dry-matter contents of microorganisms: Conversion of a measured biovolume into biomass. - Applied and Environmental Microbiology **45**: 1188–1195.

Balser, T. et al. 2005. Using lipid analysis and hyphal length to quantify AM and saprotrophic fungal abundance along a soil chronosequence. - Soil Biology and Biochemistry 37: 601-604.

Barns, S. M. et al. 1999. Wide distribution and diversity of members of the bacterial kingdom Acidobacterium in the environment. - Applied and Environmental Microbiology **65**: 1731–1737.

Bligh, E. G. and Dyer, W. J. 1959. A rapid method of total lipid extraction and purification. - Canadian Journal of Biochemical Physiology **37:** 911–917.

Bryan, A. K. et al. 2010. Measurement of mass, density, and volume during the cell cycle of yeast. - Proceedings of the National Academy of Science USA 107: 999–1004.

Edwards, U. et al. 1989. Isolation and direct complete determination of entire genes. - Nucleic Acids Research 17: 7843-7853.

Eisenhauer, N. et al. 2008. Efficiency of two widespread non-destructive extraction methods under dry soil conditions for different ecological earthworm groups. - European Journal of Soil Biology 44: 141-145.

Frostegård, A. and Bååth, E. 1996. The use of fatty acid analysis to estimate bacterial and fungal biomass in soil. - Biology and Fertility of Soils 22, 59-65.

Griffiths, R. I. et al. 2000. Rapid method for coextraction of DNA and RNA from natural environments for analysis of ribosomal DNA- and rRNA-based microbial community composition. - Applied and Environmental Microbiology 66: 5488–5491.

Hedley, M. J. et al. 1982. Changes in inorganic and organic soil phosphorus fractions induced by cultivation practices and by laboratory incubations. - Soil Science Society of America Journal 46: 970-976.

Henckel, T. et al. 1999. Molecular analyses of the methane-oxidizing microbial community in rice field soil by targeting the genes of the 16S rRNA, particulate methane monooxygenase, and methanol dehydrogenase. - Applied and Environmental Microbiology 65: 1980–1990.

Hill, T. C. J. et al. 1989. Lipid analysis in microbial Ecology: Quantitative approaches to the study of microbial communities. - BioScience 39: 535-541.

Houba, V. J. G. et al. 1986. Comparison of soil extraction by 0.01 M CaCl_2_, by EUF and by some conventional extraction procedures. - Plant and Soil **96**: 433–437.

Kempson, D. et al. 1963. A new extractor for woodland litter. - Pedobiologia 3: 1–21.

Kuo, K. 1996. Phosphorus. In: D.L. Sparks (ed.) Methods of soil analysis. Part 3, SSSA Book Series **No. 5**, Soil Science Society of America and American Society of Agronomy, Madison, pp. 869–919.

Lane, D. J. 1991. 16S/23S rRNA sequencing. In: Stackebrandt, E., Goodfellow, M. (eds). Nucleic acid techniques in bacterial systematics. John Wiley & Sons: New York, pp 115–175.

Murphy, J. and Riley, J. P. 1962. A modified single solution method for the determination of phosphate in natural waters. - Analytica et Chimica Acta 27: 31-36.

Muyzer, G. et al. 1993. Profiling of complex microbial populations by denaturing gradient gel electrophoresis analysis of polymerase chain reactionamplified genes coding for 16S rRNA. - Applied and Environmental Microbiology 59: 695–700.

Muyzer, G. et al. 1995. Phylogenetic relationships of Thiomicrospira species and their identification in deep-sea hydrothermal vent samples by denaturing gradient gel electrophoresis of 16S rDNA fragments. - Archives of Microbiology 164: 165–172.

Olsen, S. R. et al. 1954. Estimation of available phosphorus in soils by extraction with sodium bicarbonate. USDA Circ. 939. USDA, Washington, DC.

Riens, B. et al. 1991. Amino acid and sucrose content determined in the cytosolic, chloroplastic, and vacuolar compartments and in the phloem sap of spinach leaves. - Plant Physiology 97: 227-233.

Ritalahti, K. M. et al. 2006. Quantitative PCR targeting 16S rRNA and reductive dehalogenase genes for monitoring Dehalococcoides population dynamics. - Applied and Environmental Microbiology 72: 2765–2774.

Schlichting, E. and Blume H. P. 1966. Bodenkundliches Praktikum. Verlag Paul Parey, Hamburg, Berlin.

Schulze, W. X. et al. 2005a. A proteomic fingerprint of dissolved organic carbon and soil particles. - Oecologia 142: 335-343.

Schulze, W. X. 2005b. Protein analysis of dissolved organic matter: What proteins from organic debris, soil leachate and surfacd water can tell us – a perspective. - Biogeosciences 2: 75-86.

Van Veen, J. A. and Paul, E. A. 1979. Conversion of biovolume measurements of soil organisms, grown under various moisture tensions, to biomass and their nutrient content. - Applied and Environmental Microbiology **37**: 686–692.

Vestal, J. R. and White, D. C. 1989. Lipid analysis in microbial Ecology: Quantitative approaches to the study of microbial communities. - BioScience 39: 535-541.

Wilkinson, S. C. et al. 2002. PLFA profiles of microbial communities in decomposing conifer litters subject to moisture stress. - Soil Biology and Biochemistry 34: 189-200.

Yurkov AM, Kemler M, Begerow D 2011. Species accumulation curves and incidence-based species richness estimators to appraise the diversity of cultivable yeasts from beech forest soils. PLoS ONE 6: e23671.

Zelles, L. et al. 1992. Signature fatty acids in phospholipids and lipopolysaccharides as indicators of microbial biomass and community structure in agricultural soils. - Soil Biology and Biochemistry 24: 317-323.
